# A Review of Working Memory Training in the Management of Attention Deficit Hyperactivity Disorder

**DOI:** 10.3389/fnbeh.2021.686873

**Published:** 2021-07-21

**Authors:** Maha Saleh Habsan Al-Saad, Basma Al-Jabri, Abeer F. Almarzouki

**Affiliations:** ^1^Faculty of Medicine, Department of Clinical Physiology, King Abdulaziz University, Jeddah, Saudi Arabia; ^2^Public Health Sector, General Directorate of Health Affairs in Najran, Ministry of Health, Najran, Saudi Arabia; ^3^Faculty of Medicine, Department of Pediatrics, King Abdulaziz University, Jeddah, Saudi Arabia

**Keywords:** working memory training (WMT), working memory (WM), ADHD (attention deficit hyperactivity disorder), computerised cognitive training (CCT), neurodevelopmental disorders

## Abstract

Attention deficit hyperactivity disorder (ADHD) is one of the most common neurodevelopmental disorders among children. Working memory deficits underlie many of the behavioural symptoms of ADHD. Alongside psychostimulant medications, strategies to improve working memory may play an important adjuvant role in the management of ADHD. In this study, we review the role of working memory deficits in ADHD, the evidence surrounding working memory training strategies in the management of the condition, and the factors affecting the success of these strategies in alleviating ADHD symptoms. More specifically, we review several non-pharmacological interventions that target working memory deficits in ADHD, with special emphasis on cognitive working memory training. We conclude that the development of evidence-based interventions such as computerised cognitive training (CCT) could provide an alternative or adjunct to the use of psychostimulants, especially in cases where side effects are a major issue.

## Introduction

Attention deficit hyperactivity disorder (ADHD) is one of the most common neurodevelopmental disorders among children (Benyakorn et al., 2016). It is a chronic disorder with a complex and heterogeneous clinical presentation (Kofler et al., 2019), including age-inappropriate and impairing levels of inattention, hyperactivity, and impulsivity (Franke et al., 2018).

The worldwide prevalence of ADHD among children and adolescents is 7.2% (Wolraich et al., 2019) and among adults is between 2% and 5% (Polyzoi et al., 2018). Different etiological factors and numerous prenatal risk factors are associated with ADHD, including maternal substance use, stress during pregnancy, prematurity, low birth weight, and several complications of pregnancy, labour, delivery, and infancy (Sciberras et al., 2017).

ADHD affects children of both sexes, but males are diagnosed with ADHD more frequently than females, with a male to female ratio of 2–4:1 (Wolraich et al., 2019). These differences may be attributable to hyperactive behaviour being more apparent in males (Wolraich et al., 2019). Conversely, females are more prone to receive a diagnosis of the inattentive subtype of ADHD (Magnus et al., 2021). Although the disorder is classically thought of as a developmental disorder, most children diagnosed with ADHD will continue to experience symptoms during adolescence and into adulthood (Gallo and Posner, 2016). Persistent ADHD is associated with multiple negative consequences including academic underachievement, substance abuse, risky sexual behaviour, car accidents and injuries, unstable peer relationships (Bélanger et al., 2018), decreased community functioning, unemployment, and reduced income (Holbrook et al., 2016; Danielson et al., 2018).

Additionally, ADHD may present with other comorbid disorders including anxiety (Lopez et al., 2018), depression (Lopez et al., 2018), autism spectrum disorders (ASD), oppositional defiant disorder (ODD), and conduct disorder (Franke et al., 2018). Mortality is also high in people with ADHD (Bélanger et al., 2018).

The diagnosis of ADHD remains challenging due to a lack of symptom specificity, a broad list of differential diagnoses, and the presence of comorbidities (Bélanger et al., 2018). For these reasons, a comprehensive and detailed clinical assessment is particularly important (Bélanger et al., 2018).

According to the Diagnostic and Statistical Manual of Mental Disorders (DSM-5), there are three types of ADHD: predominantly inattentive, predominantly hyperactive/impulsive, and the combined type (Lopez et al., 2018). A similar definition was recommended by the International Classification of Diseases (ICD-10). There are differences in the age of onset and the necessary number of symptoms for diagnosis (Lopez et al., 2018). To diagnose a child with ADHD, the child must present with six out of the nine symptoms on the two sets of core domains (inattention and hyperactivity/impulsivity) as described in DSM-5. In youths and adults, only five of these symptoms are necessary for diagnosis. The symptoms must be observed for at least 6 months and cause substantial impairment in social, academic, and occupational performance in two or more different settings (e.g., home and school).

The age of onset of ADHD symptoms was raised from 7 years (DSM-4) to 12 years (DSM-5) to allow further flexibility in diagnosing older adolescents and adults (Wolraich et al., 2019). The symptoms of inattention include an inability to maintain focus on details, making imprudent mistakes, an inability to stay focused on duties, appearing to not listen when being spoken to, an inability to adhere to directions or arrange tasks, keeping away from duties that need mental exertion, losing important things, getting preoccupied with external stimuli, and being careless in everyday activities. The symptoms of hyperactivity/impulsivity include: squirming, feeling as if being continuously driven by an “inward engine,” an inability to stay seated when required, jumping on things, being noisy, exclaiming answers, blabbering, an inability to wait their turn, and a tendency to hinder or interrupt others. Because each of these symptoms has a different underlying neurological substrate, it is possible that different neurobiological mechanisms contribute to the clinical features of ADHD (Table 1).

**Table 1:** Typical ADHD symptoms and underlying structures.

Symptoms	Structures
Inattention	The frontal cortex, cingulate, parietal lobe, limbic system, reticular activating system, basal ganglia
Hyperactivity/Impulsivity	Premotor/motor areas, cerebellum, basal ganglia, cingulo-opercular regions, caudate regions, reticular activating system

Medications are essential for the management of patients with ADHD (Sonuga-Barke et al., 2014). Psychostimulants are considered the first-line pharmacological treatment option for ADHD (Lambez et al., 2020), with methylphenidate being the most prescribed psychostimulant. Methylphenidate can enhance cognitive function in patients with ADHD and improve cerebral cortex activity by increasing the availability of catecholamines, which play a critical role in cognitive functioning (Farr et al., 2014). However, the use of stimulants increases the risk of anorexia, weight loss, and insomnia (Briars and Todd, 2016).

Non-pharmacological interventions have also been investigated for improving cognitive function in ADHD (Sharma et al., 2015). Dietary supplementation with minerals and Omega-3 resulted in modest improvements in ADHD behavioural symptoms and emotional lability (Sharma et al., 2015). Meditation-based practices such as yoga and mindfulness are commonly practised in patients with ADHD (Sharma et al., 2015). Yoga has been found to modulate the activity of the autonomic nervous system and induce parasympathetic activity and thus improve anxiety and mood. It also reduces impulsive behaviour (Sharma et al., 2015). Mindfulness increases the density of grey matter in areas associated with memory, emotion control, and learning (Sharma et al., 2015). However, there are substantial differences between studies in the methods and the targeted ADHD subtypes and deficits, and hence these conclusions remain to be corroborated (Sharma et al., 2015).

Another promising non-pharmacological approach is neurofeedback (NFB; Ros et al., 2014). It improves self-control by using a brain-computer interface and improves behavioural symptoms of ADHD for up to 1 year after treatment (Van Doren et al., 2019). NFB training also improves visual and auditory short-term memory and auditory working memory (Nesayan et al., 2019).

Cognitive-behavioural therapy (CBT) is another non-pharmacological approach that can be implemented on an individual or group basis, or for parental education (Shabanpour et al., 2017). Previous studies have shown that CBT reduces the behavioural symptoms of ADHD (Shabanpour et al., 2017). In addition, there is evidence for other non-invasive, non-pharmacological interventions that can be offered to ADHD patients such as physical exercise, transcranial direct current stimulation (tDCS), and transcranial magnetic stimulation (TMS) to improve the behavioural and cognitive domains of ADHD (Lambez et al., 2020). However, there are relatively few studies in this field (Lambez et al., 2020).

The combination of non-pharmacological and pharmacological interventions seems promising for achieving improved cognitive function with lower medication doses, thus potentially reducing side effects. These non-pharmacological interventions target cognitive domains considered central to the cognitive deficits in ADHD, most notably working memory. Moreover, these interventions provide benefits beyond what is achievable through medication alone (Holmes et al., 2010; Catalá-López et al., 2017). Thus, training exercises targeting working memory are a promising adjunct treatment option for people with ADHD.

In this review, we provide a review of working memory and working memory training and their role in the management of ADHD. We provide an overview of the mechanisms underlying the therapeutic benefit of working memory training and identify novel directions for research to improve ADHD treatment protocols.

## Working Memory

Working memory refers to the active mental workspace that can briefly hold and manipulate information (Fang et al., 2016). Working memory capacity determines the rate and level of learning and predicts performance on mental tasks such as reading comprehension, language acquisition, reasoning, and problem-solving (Fang et al., 2016; Emch et al., 2019).

Working memory is a hierarchical process that connects detailed sensory representations to specific behavioural responses. These are linked *via* intermediate task-relevant representations and action plans in a network of different brain areas (Christophel et al., 2017).

Working memory plays a fundamental role in cognition, allowing one to hold information “in mind.” Working memory is defined by its flexibility: people are capable of storing, at least temporarily, any information. According to Baddeley’s multi-element model of working memory, considered the predominant hypothetical model (Baddeley, 2007), the working memory system includes three anatomically and functionally discrete elements:

1. The visuospatial sketchpad, that stores visual and spatial material with limited capacity (Emch et al., 2019).
2. The phonological loop, which has two components. The first component, the phonological store, holds visually presented and auditory-verbal information that can be kept active in the second component, the articulatory loop, through subvocal rehearsal (Emch et al., 2019).
3. The central executive, which allocates the attentional resources for the organisation, deeper processing, and storage of different types of information elements and is considered the master element of working memory (Emch et al., 2019).

The episodic buffer integrates information from the phonological loop, visuospatial sketchpad, and long-term memory (Emch et al., 2019). The buffer is a passive system with limited capacity, believed to be linked to long-term memory and semantic meaning (Baddeley, 2017). Its main function is to link information across different domains to form integrated elements of visual, spatial, and verbal information that are ordered in an episodic and chronological manner.

Working memory includes multiple stages: encoding, maintenance, and retrieval, as well as some process of attention regulation that resists interruption by irrelevant information (Emch et al., 2019).

Encoding is the initial process of perceiving and learning information. Working memory stores information for immediate or long-term use (long-term memory). Encoding can be visual (converting images and visual sensory information to memory), elaborative (relating new information to previously stored knowledge), semantic (processing and encoding sensory input that has a particular meaning), acoustic (encoding auditory inputs), or other (tactile, odours, tastes; Baddeley, 2017).

Maintenance (or storage) of information is the process of placing the acquired information into memory. Memory can be stored in short-term or long-term memory, with the former being a component of working memory. Short-term memory is only used to refer to the storage of information for a short while and working memory refers to the components of memory that uses the information to manipulate this information (Baddeley, 2017).

Retrieval is the mental process of recalling information that was previously stored. There are three main types of recall: free, cued, and serial. Free recall occurs when individuals are asked to recall items previously presented on a list. Cued recall is when a person receives a list of items to remember and is then offered cues to help them recall those items during testing. Serial recall refers to recalling events or items in the order in which they occurred (Baddeley, 2017).

## Neural Correlates of Working Memory

Early studies that used resting-state functional magnetic imaging (fMRI) have shown that large-scale brain regions exhibit high-amplitude fluctuations, which are enhanced during rest and reduced during cognitive tasks (Konrad and Eickhoff, 2010; Castellanos and Proal, 2012). This intrinsic functional inter-neuronal connection represents the brain’s physiological reference and the so-called default mode network (DMN; Konrad and Eickhoff, 2010; Castellanos and Proal, 2012). The DMN involves the anterior medial prefrontal cortex (amPFC), the posterior cingulate cortex (PCC), the medial temporal lobe (MTL) subsystem, and the dorsomedial PFC (dmPFC) subsystem (Castellanos and Proal, 2012).

The executive control network (ECN), also known as the frontoparietal network, includes the dorsolateral PFC (dlPFC), anterior PFC (aPFC), anterior cingulate cortex (ACC), lateral frontal pole, lateral cerebellum, anterior insula, caudate, and inferior parietal lobe (Konrad and Eickhoff, 2010; Castellanos and Proal, 2012). This circuit has been defined as the task-positive circuit as it shows more activity during tasks than during rest (Konrad and Eickhoff, 2010). It also directs decision making by incorporating exterior stimuli with the corresponding interior representations (Castellanos and Proal, 2012). The DMN and ECN are inversely correlated as the activation of the ECN is associated with lower activity of the DMN and *vice versa* (Konrad and Eickhoff, 2010).

The dorsal attentional network includes the intraparietal sulcus and frontal eye fields, which are essential in attention shifting and control (Castellanos and Proal, 2012). The ventral attentional network, also known as the salience network (SN), involves the fronto-insular cortex (FIC), temporoparietal junction and supramarginal gyrus (Castellanos and Proal, 2012). The visual network includes the visual cortex and lateral temporal region MT+, which is linked to DAN *via* the superior parietal lobule and intraparietal cortex (IPC). MT+ also is connected with frontal areas (Castellanos and Proal, 2012). The occipital cortex, which contains most of the visual cortex regions, interacts with DAN to hold attention and suppress attention to the distractor (Castellanos and Proal, 2012). The motor network involves simultaneous spontaneous low-amplitude fluctuations between the supplementary motor cortex, primary motor cortex, primary and secondary sensory cortex, putamen, cerebellum, thalamus, and ventral premotor cortex (Castellanos and Proal, 2012). These fundamental networks can be investigated in ADHD and other neurocognitive disorders (Castellanos and Proal, 2012).

Functional neuroimaging studies have demonstrated that working memory is related to the prefrontal cortex (PFC), inferior and middle temporal lobes, and zones close to the IPC (Fang et al., 2016). Similarly, these regions are linked to cognitive function, PLL, declarative memory, and episodic processing (Fang et al., 2016). Moreover, working memory task-based fMRI studies have shown that information encoding and manipulation is related to dorsolateral PFC (dlPFC). Error recognition and execution adjustment, on the other hand, are related to activity in the dorsal ACC (dACC), which is considered to be the attention organiser (Chai et al., 2018). Information selection, retrieval, and inhibition regulation are linked to neurons extending from the ventrolateral PFC (vlPFC) to the anterior insula (Fang et al., 2016). The left PFC and the right PFC might be primarily associated with verbal working memory and spatial working memory, respectively, as indicated in previous meta-analyses (Emch et al., 2019). However, there is no common agreement on the functional organisation of this brain region (Emch et al., 2019).

fMRI studies have also proposed that the articulatory loop is linked to Broca’s area, premotor cortex (BA6), supplementary motor area, and insula on the left hemisphere. The phonological store is linked to BA 40, relating to the inferior parietal lobule in the left hemisphere (Emch et al., 2019). Hence these areas are critical for all kinds of visual working memory tasks (Emch et al., 2019). The cerebellum has been suggested to be involved in subvocal rehearsal (Emch et al., 2019). Similarly, the basal ganglia (BG) are essential brain structures involved in motor control, facilitating appropriate motor behaviour and inhibiting inappropriate motor behaviour (Emch et al., 2019). The BG is also involved in working memory and language production (Emch et al., 2019). Furthermore, visual working memory is related to parts of the limbic system such as the cingulate (Emch et al., 2019). However, the contribution of the cerebellum, BG, and limbic system to working memory has long been undervalued (Emch et al., 2019).

In addition, studies that used resting-state fMRI have revealed that working memory functioning is related to resting-state neuronal activity (Fang et al., 2016). For example, Hampson et al. (2010), reported that working memory accuracy was related to the coherent neuronal interconnection between the dlPFC and medial PFC (Hampson et al., 2010; Fang et al., 2016). However, such complex cognitive functions are characterised by cooperation between multiple brain areas rather than being driven by one or two regions (Fang et al., 2016). Furthermore, the individual disparities in working memory are related to the efficient functional connection from dlPFC to Dacc and from the right dlPFC to the left FIC. The high sensitivity of left FIC to inputs from dlPFC assists in efficient manipulation of information and hence improved working memory functioning during working memory tasks (Fang et al., 2016).

Moreover, diffusion MRI in healthy individuals has also demonstrated that working memory capacity is linked to a corticocortical pathway between the frontal and parietal regions. In addition, the updating of working memory information included a subcortical neural pathway linking between frontal and parietal regions through the thalamus and BG (Ekman et al., 2016). Moreover, working memory capacity is directly correlated to the integrity of white matter in frontal and parietal regions on diffusion MRI (Ekman et al., 2016).

## Cognitive Deficits in ADHD

Although many cognitive targets have been investigated in ADHD (see Table 2), this review focuses on working memory. Working memory deficits are an important potential endophenotype of ADHD (Kasper et al., 2012; Chacko et al., 2013). The earliest model of the neuropsychological correlate of ADHD was the prefrontal-striatal-cerebellar model (Castellanos and Proal, 2012).

**Table 2:** Examples of commonly investigated cognitive tests in ADHD.

References	Age range (years)	Neuropsychological outcomes	Academic abilities
Klingberg et al. (2005)	7–12	DS→verbal working memory BS→visual working memory Stroop test→inhibition Conner’s Parent Rating Scale and DSM-IV→ADHD core symptoms	None
Holmes et al. (2010)	9–10	AWMA→working memory	WART→reading WOND→mathematical reasoning
Johnstone et al. (2010)	8–12	CS→verbal working memory GO NO GO, RT→inhibition Add ball task→attention	None
Gray et al. (2012)	12–17	DSB→verbal working memory SS from CANTAB→visual working memory D2 test total →attention	WART
Green et al. (2012)	7–14	WISC-VI→verbal working memory and processing speed	None
Pan et al. (2016)	6–12	Stroop word colour→inhibition	None

*Abbreviation: DS, digit span; BS, board span; AWMA, automated working memory assessment; WART, wide range achievement test; WOND, Weschler objective numerical dimensions; BRIEF, Behavior rating inventory of executive function; DSB, digit span backwards; SS, spatial span; CANTAB, Cambridge Neuropsychological Test Automated Battery; CS, counting span; RT, reaction time; WISC-VI, Weschler intelligence scale for children 4th edition*.

### Animal Studies

Animal models of ADHD show good predictive validity that allows the assessment and development of new therapeutic interventions. For example, they have shown polymorphism in several genes related to catecholaminergic neurotransmission. These include the dopamine transporter (DAT), dopamine D4 receptor (DRD4), and dopamine beta-hydroxylase genes. A lack of DAT results in decreased release of dopamine from the nerve terminal, which is accompanied by a fivefold increase in the concentration of extracellular dopamine. On the presynaptic side, mRNA and D1 and D2 receptor protein levels in the BG decrease. Compounds such as amphetamine, methylphenidate, and cocaine have a direct action on DATs and inhibit hyperactivity (Rahi and Kumar, 2021). While studies with transgenic mice have provided valuable information on the neurobiological factors underlying ADHD, no single gene or transgenic animal model represents the entire ADHD spectrum. Therefore, complex gene-gene as well gene-environment interactions must also be taken into account.

### Human Studies

Recent studies propose that working memory deficits occur in approximately 80–85% of children with ADHD when evaluated with cognitive tasks (Coghill et al., 2014; Karalunas et al., 2017; Kofler et al., 2018a, 2020). Studies have also shown that children with ADHD have more working memory deficits than typically developing children (Kasper et al., 2012). Several studies have shown that children diagnosed with ADHD are impaired in all working memory elements, with the most significant impairment found in the central executive, resulting in an inability to focus on a task (Kofler et al., 2010).

Working memory deficits have been linked with inattention (Kofler et al., 2010), hyperactivity (Hudec et al., 2015), and impulsivity (Raiker et al., 2012). They have been associated with ADHD-related impairment in academics (Friedman et al., 2018), organisational (Kofler et al., 2018b), social (Bunford et al., 2015), and family life (Kofler et al., 2017). Longitudinal studies show that less severe working memory deficits are associated with lower symptom severity (Halperin et al., 2008; van Lieshout et al., 2016; Salari et al., 2017), and reductions in ADHD symptoms with age seem to be limited to a subset of children who show improvements in working memory over time (Karalunas et al., 2017). These studies highlight the significant influence that working memory has on the symptomatology of many children with ADHD and have motivated a recent surge in studies aiming to enhance working memory in children with ADHD (Kofler et al., 2018a).

## Neural Correlates of ADHD

Functional neuroimaging studies using specific or multiple cognitive tasks in ADHD patients have found widespread multiregional dysfunctions. These include the lateral PFC and its connection to the BG, medio- and orbitofrontal regions, and the cingulate cortex. In addition, dissociation in neural connectivity in the frontoparietal, fronto-limbic and fronto-cerebellar networks have also been observed.

A meta-analysis of fMRI studies that were conducted on ADHD patients (*n* = 111) and controls (*n* = 113), revealed a reduction in neural activity in superior and middle PFC in both hemispheres, as well as the medial frontal cortex and ACC in the left hemisphere. A recent functional MRI study investigated more than 100 children and adults with ADHD using a visual-spatial working memory task. It showed two separate effects according to working memory demand: enhancement of neural activity in the inferior prefrontal cortex (IFC) under high working memory demand and a reduction in neural activity in IFC under low working memory demand (Rubia, 2018).

## Working Memory Training

Working memory training aims to improve working memory through a series of tasks that help the trainee engage and practice this cognitive function. Prevalent are computerised cognitive training (CCT) interventions, which can provide training in a wide range of cognitive tasks (e.g., working memory, attention, inhibitory control) in multiple training sessions over several weeks, often in a game format design (Table 3). The duration and number of sessions differ according to each specific implemented program (Veloso et al., 2019). CCT is described as adaptive when the level of task difficulty is automatically adapted to the user’s performance, and the training sessions can be accomplished in any preferred setting (e.g., a clinic, home, or school; Sonuga-Barke et al., 2014).

**Table 3:** Common cognitive interventions.

Cognitive Test	Target
Cogmed	Visuospatial and spatioverbal working memory
REMINDER	Memory Storage and recall strategy
Captain’s Log	Attention, working memory, visuomemory function
CogniPlus	Attention, working memory, visuomotor function, long-term memory
Locu Tour	
Pay Attention!	Attention
RehaCom	Attention, memory, executive functions

Available CCT products that include working memory training are listed in Table 4 and include the Cogmed Working Memory Training (Klingberg et al., 2005; Beck et al., 2010; Green et al., 2012; Chacko et al., 2013; Egeland et al., 2013; Hovik et al., 2013; van Dongen-Boomsma et al., 2014; van der Donk et al., 2015; Bigorra et al., 2016), BrainTrain (Steiner et al., 2014), Braingame Brian (van der Oord et al., 2014), CogniPlus (Minder et al., 2018), Activate (Sinnari et al., 2019), Project: Evo (Davis et al., 2018), Attention Pay (van der Donk et al., 2015), Persian software (Azami et al., 2016), Lumosity (Azami et al., 2016), and Captain’s log (Rabiner et al., 2010). These products have similar goals but differ in the extent to which they include elements like acquiring points, providing feedback, representing skill progression, accumulating rewards, and framing the training within a narrative context (Oldrati et al., 2020).

**Table 4:** CCT Products.

CCT Products	Website
Cogmed Working	https://www.cogmed.com/
Memory Training
BrainTrain	https://www.braintrain.com/
Braingame Brian	http://en.gamingandtraining.nl/description-braingame-brian/
CogniPlus	https://www.schuhfried.com/cogniplus/
Activate	https://www.additudemag.com/treatment/activate/
Project: Evo	https://projectevo.org/
Attention Pay	-
Persian Software	-
Lumosity	https://www.lumosity.com/en/
Captain’s Log	https://www.braintrain.com/captains-log-mindpower-builder/

The Cogmed Working Memory Training program (CWMT, Pearson, UK) has become the most popular and widely studied CCT program, and it has both supporters and detractors (Shinaver et al., 2014; Sonuga-Barke et al., 2014). The program consists of sessions of working memory tasks in the form of simple games on a computer or tablet. The training sessions take around 45–50 min to complete, 5 days per week, over 5 weeks, with weekly rewards. The complexity of the tasks is automatically adjusted based on the person’s performance.

## Effects of Working Memory Training

**Figure 1:**
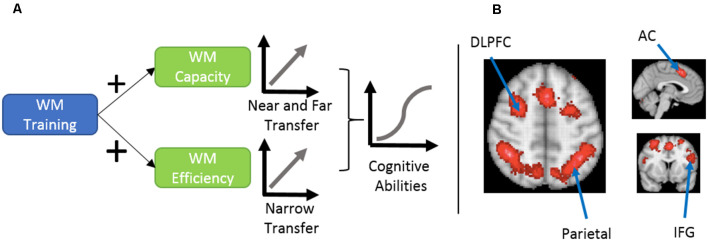
**(A)** Proposed mechanism of cognitive ability enhancement using working memory training, as explained by von Bastian et al. (2013). **(B)** An association-test map displaying brain regions that were consistently reported in 1091 studies investigating working memory. Working memory training engages executive and dorsal attentional networks. This involves brain regions like dorsolateral prefrontal cortex (DLPFC), anterior cingulate (AC), inferior frontal gyrus (IFG), and parietal regions. Map prepared using Neurosynth meta-analysis of the term working memory (Yarkoni et al., 2011). Similar neural networks show decreased signals in attention deficit hyperactivity disorder (ADHD) patients performing working memory tasks.

In this section, we discuss the evidence for the efficacy of working memory training in the general population, which helps us understand its potential role in ADHD management. Figure 1 provides an overview of the potential mechanisms to improve working memory.

Two general mechanisms explain the effects of working memory training (von Bastian and Oberauer, 2014). The first is increased working memory *capacity*, which enables people to hold more items in their working memory, and the second is increased *efficiency* in using available working memory capacity.

At the behavioural level, an increase in working memory capacity results in performance improvements in tasks on which the person was not trained, but that share some variance with the training tasks (Klingberg, 2010). Given that working memory capacity significantly correlates with several cognitive abilities, these improvements should manifest in several measures which are independent of the precise materials and structure of the tasks (Schmiedek et al., 2010). In general, two terms are used to define the transfer of training: (1) near-transfer which refers to gains in abilities directly related to the training task; and (2) far-transfer, which are gains in different but related abilities.

Meiran, Dreisbach, and von Bastian noted in 2019 that while meta-analyses assessing the existence of far-transfer benefits of working memory training have found negative (Melby-Lervåg et al., 2016), limited or short-lived positive results (Au et al., 2015), performance gains on similar tasks are substantial, and trainees typically performing above average after training. For example, in one study, young adults could recall twice as many items from a list as the average healthy adult after 20 sessions of working memory training (von Bastian and Oberauer, 2014).

Understanding the neural correlates of training-induced improvements would help guide and monitor training strategies. However, this is challenging given the existence of many parallel behavioural changes that occur during working memory training (Klingberg, 2010). There are, however, studies that show a positive correlation between working memory capacity and brain activity in different task-relevant areas. For example, inter-individual differences in working memory capacity positively correlate with activity in the IPC (Gray et al., 2003; Todd and Marois, 2004; Vogel and Machizawa, 2004; Lee et al., 2006; McNab and Klingberg, 2008) and increases in working memory capacity during childhood are positively correlated with brain activity in the prefrontal cortex and intraparietal sulcus (Klingberg et al., 2002; Kwon et al., 2002; Ciesielski et al., 2006; Crone et al., 2006; Scherf et al., 2006; Olesen et al., 2007). On the other hand, the decline in working memory during ageing is associated with decreased activity in certain prefrontal areas (Rajah and D’Esposito, 2005; Persson and Nyberg, 2006).

To assess the effects of working memory training on the brain, most neuroimaging studies have relied on recording changes in the activity of brain regions during working memory task performance before and after training (Constantinidis and Klingberg, 2016). Working memory training is associated with changes in the neuronal activity of brain regions that are activated during working memory tasks before training (Constantinidis and Klingberg, 2016). This finding suggests that improvements in task performance during working memory training reflect improved working memory capacity (Constantinidis and Klingberg, 2016).

Earlier research with healthy adults using fMRI suggests that working memory training has a direct effect on the prefrontal cortex, posterior parietal cortex, and dopamine receptor binding. Studies have shown that working memory training results in enhanced neuronal activity in the prefrontal cortex and the posterior parietal cortex, which are areas linked to working memory processing (Green et al., 2012). Working memory training also increases the density of D1 receptors in these brain regions (Green et al., 2012) and increases activity in the striatum (Constantinidis and Klingberg, 2016). Although this change in striatal activity is not consistent, it is predictive of working memory capacity changes during development (Constantinidis and Klingberg, 2016).

Several studies of the brain’s functional connectivity have shown that stronger functional connectivity between frontal and parietal cortices plays a significant role in working memory improvement with training (Constantinidis and Klingberg, 2016). A study using TMS of the parietal cortex during training on higher-demand working memory tasks revealed stronger functional connectivity between the frontal cortex and the parietal cortex, and between the parietal cortex and the occipital cortex (Constantinidis and Klingberg, 2016). Furthermore, a magnetoencephalography study of functional connectivity changes during, before, and after working memory training found increased connectivity between frontal, parietal and lateral occipital cortex, which was associated with working memory improvements (Constantinidis and Klingberg, 2016).

An increase in synaptic connection and myelin density in axons connecting the involved regions might be the mechanism underlying training-related functional connectivity changes (Constantinidis and Klingberg, 2016). As previously mentioned, dopamine plays an important role during working memory training (Constantinidis and Klingberg, 2016). Positron emission tomography studies in humans and other animals have shown that working memory training results in changes in the release of dopamine as well as the density of dopamine receptors (Constantinidis and Klingberg, 2016). Changes in striatal dopamine release might also enable cortical plasticity (Constantinidis and Klingberg, 2016).

## Working Memory Training in ADHD

Several studies have provided evidence for the efficacy of working memory training in ADHD. A meta-analysis of 54 studies investigating a range of CCT programs (including most of the ones mentioned above) showed that they were associated with improvements in working memory, as well as other elements of executive functioning, in people with ADHD (Veloso et al., 2019). Importantly, the majority of studies involving longitudinal follow-up showed that these improvements were maintained over time.

The reason CWMT attracts more attention compared to most other CCT programs likely stems from the fact that its effectiveness has been established by several studies (Simons et al., 2016). Furthermore, studies investigating the effects of CWMT tend to have a better experimental design, often including active controls. These randomised controlled trials of CWMT provide strong evidence that it enhances performance on other working memory tasks that have similar processing demands. While other CCT programs have been shown to improve working memory, evidence for this from randomised controlled trials is often lacking. Furthermore, when randomised controlled trials of these interventions are conducted, they are often compared with passive control groups (Simons et al., 2016). Due to its widespread use, popularity, and strong evidence for its efficacy, the following section will focus on studies utilising CWMT.

**Table 5:** Evidence for the efficacy of Working Memory Training (WMT) in ADHD.

Study	Study design	Sample	Outcome
Veloso et al. (2019)	Systematic review	22 studies children and adolescents with ADHD	13/18 studies found improvements in executive function with cognitive training. 17/22 studies found positive transfer effects on ADHD symptoms, academic achievement, social skills, etc. 7/9 studies found that treatment effects were maintained over time.
Klingberg et al. (2005)	Randomised controlled trial	53 children with ADHD	Adaptive CWMT improved working memory, inhibition control, complex reasoning, and ADHD symptoms on the parent-rating scale. Effects were maintained post-training at 3 months follow-up.
Gray et al. (2012)	Randomised controlled trial	60 youth with ADHD (12–17 years old)	Compared to math training, CWMT improved visuospatial working memory and visual working memory, attention, math, and reading.
Holmes et al. (2010)	Intervention study of CWMT with and without ADHD medication	25 children with ADHD	Medication improved visuospatial working memory and CWMT improved visuospatial working memory, visual working memory, visuospatial short-term memory, and verbal short-term memory. The effects lasted for at least 6 months after training and were larger than the effects of medication used alone.
Beck et al. (2010)	Nonrandomised controlled trial	52 children and adolescents with ADHD	Improved working memory and reduction in inattention along with an increase in positive behaviours. Effects were still noticed by parents 4 months later.
Green et al. (2012)	Randomised controlled trial	26 children with ADHD	Improved working memory.
Rapport et al. (2013)	Meta-analysis	25 studies of children with ADHD	No evidence that cognitive training improves cognitive, behavioural, or academic abilities.
Melby-Lervåg and Hulme (2013)	Meta-analysis	23 studies of children and adults with ADHD and typically developing children and adults	Working memory training produces short-term gains in working memory skills. The gains do not persist for long periods and do not generalise to other skills.

Several studies have found significant clinical effects on executive functions in ADHD individuals following CWMT intervention (Table 5). Klingberg et al. (2005) conducted the first randomised controlled trial of CWMT in 53 children with ADHD who were randomised to either adaptive (adjusted to the user’s performance) or non-adaptive CWMT (not adjusted to the user’s performance) This research showed that adaptive CWMT resulted in significant improvement of working memory, inhibition control, complex reasoning, and a reduction in ADHD symptoms on the parent-rating scale; these effects were maintained at a post-training 3 months follow-up. Gray et al. (2012) evaluated the impact of CWMT on working memory in a sample of 60 youth who were diagnosed with ADHD and learning disability. The main finding was that CWMT improved visuospatial working memory and visual working memory, and also led to gains in attention, math, and reading.

Consistent with the findings of previous studies, Holmes et al. (2010) found that CWMT produced significant clinical effects in a sample of 25 children with ADHD, who were assessed before and after training as well as on and off medication. The researchers concluded that, although medication significantly improved visuospatial working memory, CWMT led to significant gains on multiple memory tasks such as visuospatial working memory, visual working memory, visuospatial short term memory, and verbal short term memory. The improvements lasted for at least 6 months after training and were larger than the effect of the medication alone.

In a different study, Beck et al. (2010) found that children with ADHD who underwent CWMT showed an improvement in working memory and a reduction in inattention along with an increase in positive behaviours compared to age-typical children in the wait-list group. These improvements were still noticed by their parents 4 months later. However, the study reported no positive effects on hyperactivity/impulsivity symptoms rated by their patients and teachers (Beck et al., 2010). Similarly, Green et al. (2012) reported that adaptive-CWMT in ADHD children led to significant gains in working memory tasks, but there was no effect on ADHD symptoms rated by the parents (Gray et al., 2012).

There is some evidence regarding the beneficial effects of CWMT on fluid intelligence, which may play a significant role in educational achievement (Kaufman et al., 2009). Bergman Nutley et al. (2011) found significant improvements on measures of fluid intelligence in children who trained on a non-verbal reasoning task. This finding was also replicated on working memory training tasks other than CogMed (i.e., N-Back), and lasted for at least 3 months post-training (Jaeggi et al., 2011). Söderqvist and Bergman Nutley (2015) reported that CWMT in typical learners led to higher academic achievement in math and reading 2 years after training.

Other studies have found less promising results with regards to CWMT outcomes. These studies concluded that, although CWMT led to improvements in certain aspects of working memory and executive function, these improvements failed to generalise to academic achievement (Chacko et al., 2013; Bigorra et al., 2016). Similarly, a previous meta-analysis that examined the effectiveness of cognitive training in children with ADHD concluded that there is no significant effect of training on the cognitive, behavioural, and/or academic abilities of these children (Rapport et al., 2013). However, the researchers stated that their findings might be due to methodological limitations across the reviewed studies. In line with the previous meta-analysis, Melby-Lervåg and Hulme (2013) reported that working memory training resulted in short-term gains in working memory skills, but these gains do not generalise to other skills or persist for long periods.

With regards to the transfer effects of cognitive training, it has been theorised that training-based improvements in working memory capacity and attention generalise to other functioning domains. However, the results across the literature are inconsistent (Sala et al., 2019). CWMT has shown near-transfer effects in children diagnosed with ADHD, poor working memory, and/or attention deficits (Rossignoli-Palomeque et al., 2018). Far-transfer effects were reported on measures of reasoning and inhibition (Klingberg et al., 2005) and executive function (Holmes et al., 2010; Bigorra et al., 2016). Far-transfer effects were also reported on ADHD symptoms (Beck et al., 2010; Bigorra et al., 2016) as well as on academic abilities such as math (Holmes et al., 2010; Dahlin, 2013; Egeland et al., 2013) and reading (Egeland et al., 2013). The effects of training on near-transfer were short-lived, and on far-transfer ranged from 4 to 8 months (Rossignoli-Palomeque et al., 2018).

The inconsistent results regarding the effectiveness of cognitive training on improving ADHD cognition and behaviour may reflect differences in training strategy (exact training protocols, trained populations, concomitant treatment, etc). The heterogeneity of training strategies used in previous studies may itself reflect a lack of understanding of the mechanisms that underlie the response (or lack thereof) to cognitive interventions.

## Factors Potentially Influencing The Effect of Working Memory Training

Several factors are known to influence the outcomes of CCT interventions, including characteristics of the training plan (such as the intensity, duration, adaptivity of the training task), as well as individual differences in age, cognitive abilities, biological factors, personality factors, motivational factors, and emotional factors (von Bastian and Oberauer, 2014; Barkus, 2020; Dentz et al., 2020). Awareness of the role of these factors may help optimise CCT strategies in the future.

### Characteristics of the Training Plan

Most commercially available CCT programs target a mix of different cognitive skills. While this was thought to lead to more transfer effects than targeting single skills, empirical evidence for this – in the form of comprehensive direct comparisons between these strategies – is lacking. The little evidence that is available in this regard suggests that programs that provide intensive practise of one aspect of a cognitive function like working memory are probably more effective at achieving transfer effects than those that involve multiple cognitive skills (von Bastian et al., 2013).

There is considerable heterogeneity in the CCT literature regarding the number and duration of training sessions (Luis-Ruiz et al., 2020; Wiest et al., 2020; Grinberg et al., 2021). Importantly, few studies have attempted to directly determine the optimal length and intensity of CCT. Nonetheless, several studies have shown that the effect of such interventions is dose-dependent, meaning the length and intensity of these programs influence outcomes (Jaeggi et al., 2008; Alloway et al., 2013). Although many commercially available CCT products implement this, the influence of adapting to the individual trainee’s performance to maintain a level of difficulty that is challenging on performance gains is also controversial (von Bastian and Oberauer, 2014).

### Characteristics of the Trainee

Several factors potentially contribute to the relatively high between-person variability in performance gains and transfer effects following working memory training. Working memory training programs tend to generally be more effective in younger than older individuals, with evidence suggesting that the relationship between age and training is linear throughout the lifespan (Wass et al., 2012; Melby-Lervåg and Hulme, 2013). Characteristics such as intrinsic motivation (finding enjoyment or satisfaction in engaging in a particular behaviour) are known to correlate with working memory performance (Brose et al., 2010; Duckworth et al., 2011). Whether or not such characteristics directly influence the performance gains following CCT interventions has not yet been established. On the other hand, personality traits like neuroticism (related to higher excitability and emotional responsiveness) are associated with lower performance gains after working memory training (Studer-Luethi et al., 2012).

Twin studies show an estimated heritability of working memory capacity of around 50% (Ando et al., 2001). As previously mentioned, the importance of dopamine in this context is evidenced by the finding that working memory training increases dopaminergic receptor density and dopaminergic pathway activity (Green et al., 2012; Constantinidis and Klingberg, 2016). In addition, working memory performance appears to be significantly influenced by dopamine-relevant genes (Bäckman and Nyberg, 2013). Carriers of the DAT1 9/10-repeat allele benefit more from working memory training compared to carriers of the DAT1 10-repeat allele (Brehmer et al., 2012). This difference may be explained by the fact that 10-repeat carriers have increased gene expression, which leads to a higher level of dopamine reuptake, and, consequently, fewer active dopaminergic pathways available (Swanson et al., 2000).

Another genetic factor that contributes to the availability of dopamine is the allelic variations in the LIM homeobox transcription factor 1 alpha (LMX1A; Nakatani et al., 2010). A study conducted by Colzato et al. (2011) showed that two single nucleotide polymorphisms (SNP) that influence the number of dopamine neurons in the midbrain have a significant relationship with verbal working memory training.

Finally, brain-derived neurotrophic factor (BDNF), which is involved in hippocampal plasticity, may also have a role in working memory training (Loprinzi and Frith, 2019). One example of this is the Val^66^ Met SNP in the BDNF gene. In comparison to Val homozygotes, carriers with the Met allele perform poorer in certain memory tasks (Hariri et al., 2003) and have reduced hippocampal volume (Bueller et al., 2006). A comparison of Val/Val homozygotes with carriers of the Met allele (Colzato et al., 2011) showed that, while both groups improved during CCT, only the Val/Val homozygous individuals showed far-transfer to a divided attention task.

## Discussion

ADHD has a complex and heterogeneous disorder and a one-size-fits-it-all treatment approach is likely to provide limited results in many cases. The present study provides a review of the neurocognitive mechanisms that underlie changes in working memory and how these mechanisms may influence the response to working memory training.

Cognitive training programs that target working memory are a potentially useful therapeutic option in ADHD. Response to working memory training may reflect changes in the neuronal activity of brain regions that are activated during working memory tasks before training (Constantinidis and Klingberg, 2016) and multiple factors may explain individual differences in response (Ando et al., 2001), such as the settings of the training regime and several individual factors.

Given that working memory capacity significantly correlates with several cognitive abilities, improvements in working memory capacity should manifest in several measures which are independent of the materials and structure of the tasks (Schmiedek et al., 2010; Rosenberg et al., 2020). The possibility of achieving near-transfer and far-transfer following working memory training arguably adds additional support in favour of researching and improving working memory training programs for ADHD.

Although pharmacological interventions such as stimulants provide a substantial improvement in ADHD cognitive symptoms, this comes at the cost of a higher risk of side effects. Cognitive training provides additional benefits above and beyond those of pharmacological interventions in ADHD, though additional studies of higher methodological quality comparing these two treatment strategies are necessary.

## Author Contributions

MA-S, BA-J and AA: literature search, writing, editing, reviewing. All authors contributed to the article and approved the submitted version.

## Conflict of Interest

The authors declare that the research was conducted in the absence of any commercial or financial relationships that could be construed as a potential conflict of interest.
